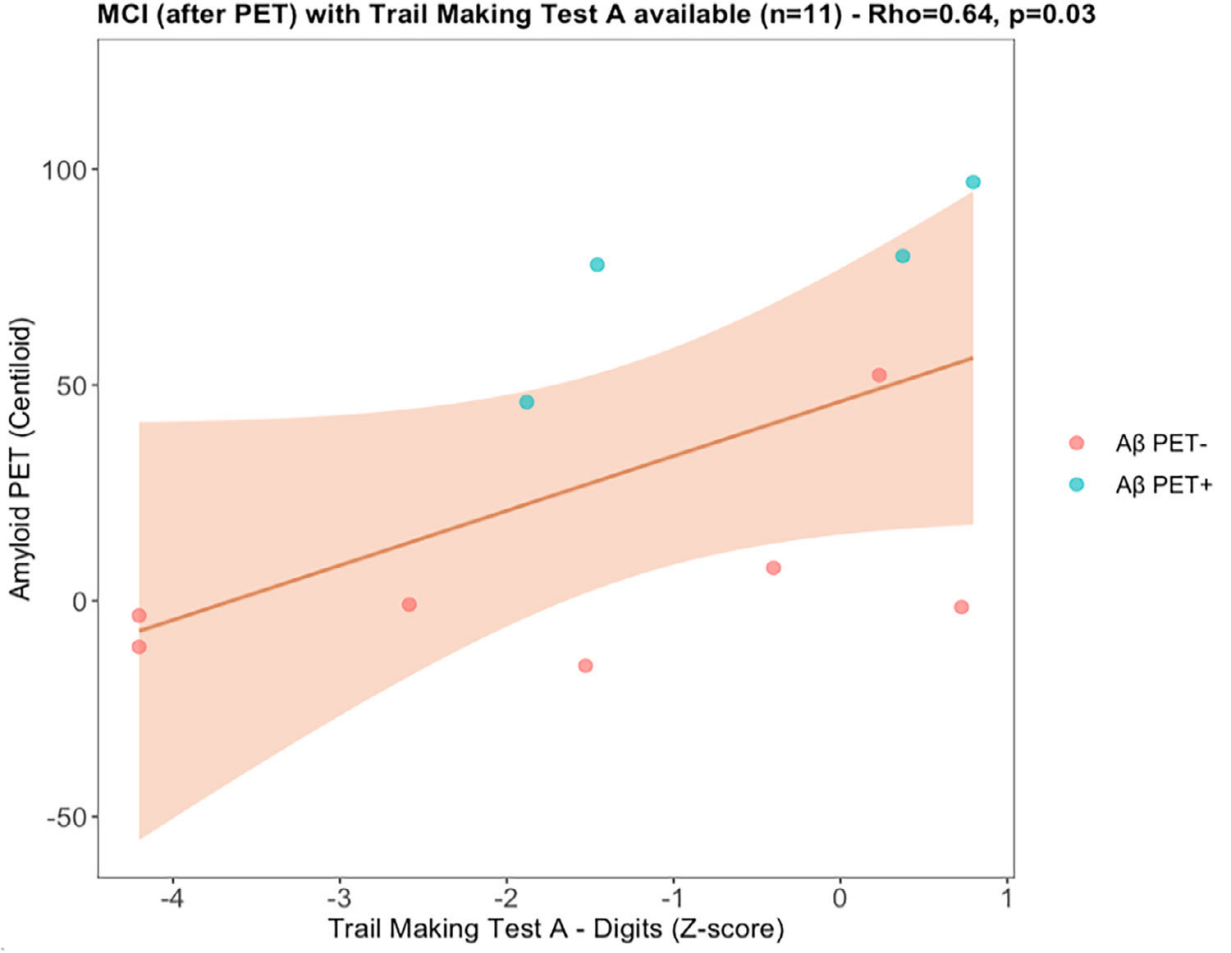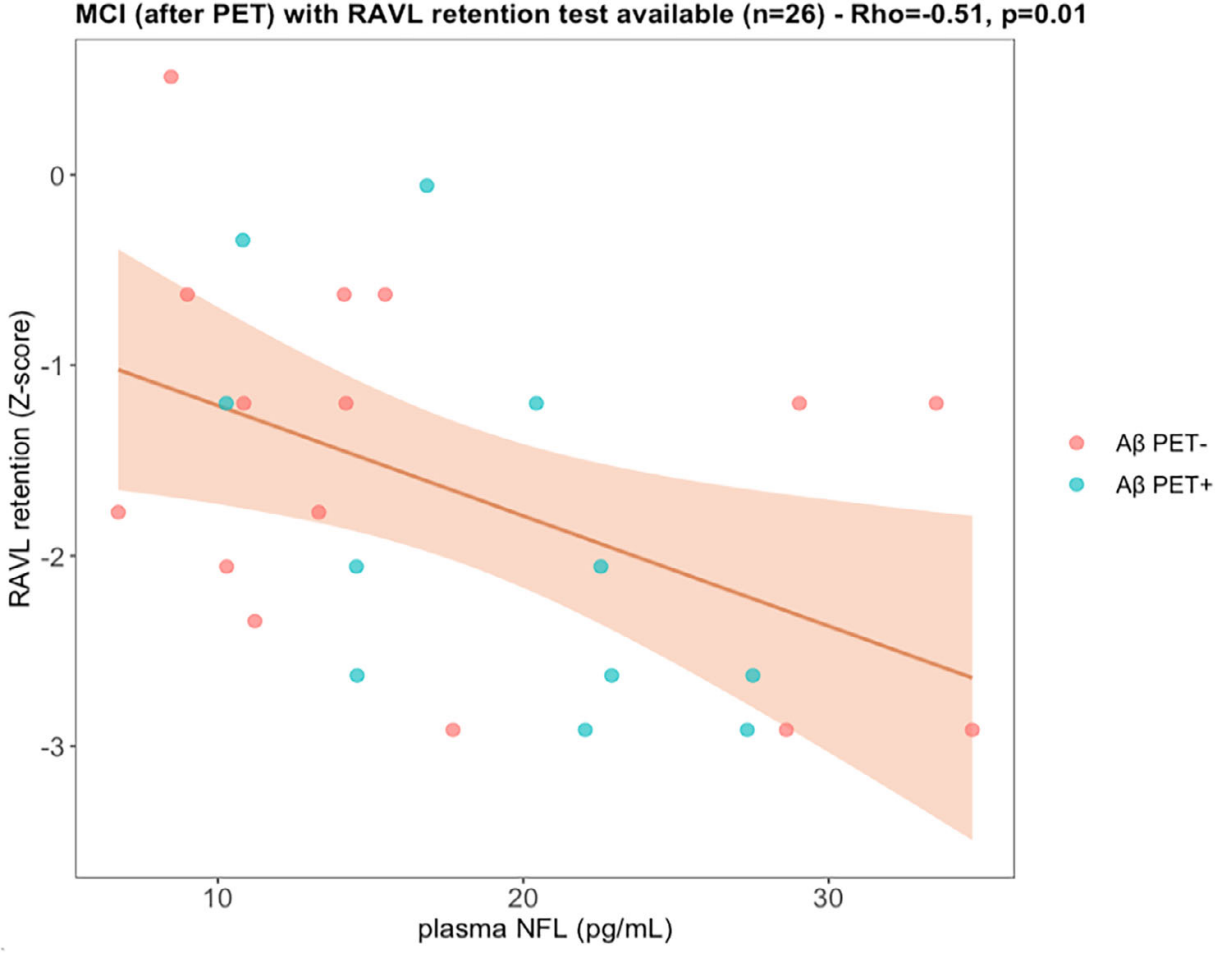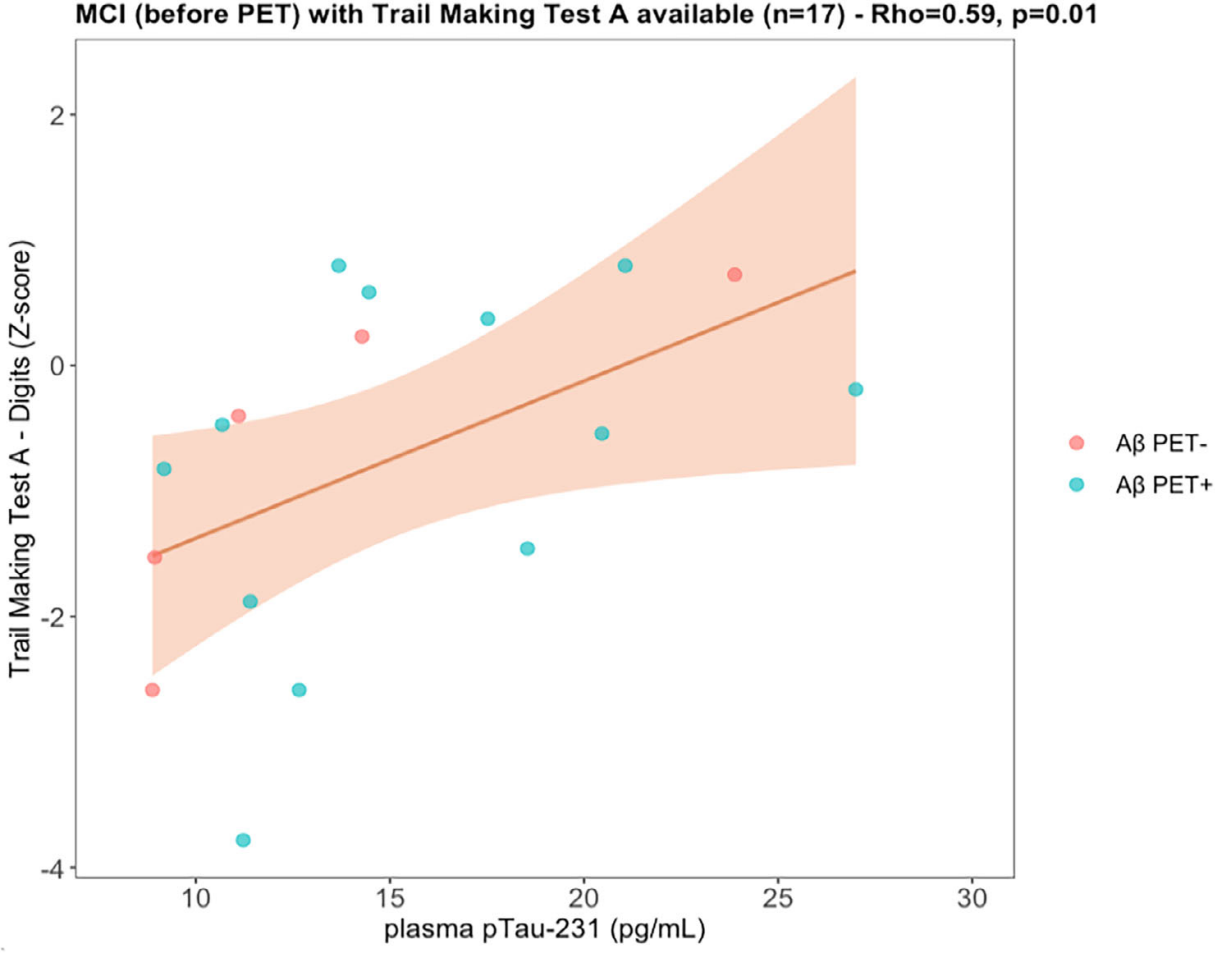# Associations between neuropsychological tests, plasma biomarkers and PET amyloid scans in a memory clinic cohort

**DOI:** 10.1002/alz.092323

**Published:** 2025-01-09

**Authors:** Marco Bucci, Ove Almkvist, Marina Bluma, Irina Savitcheva, Mariola Zapater‐Fajari, Konstantinos Chiotis, Anna Matton, Miia Kivipelto, Andreas Jeromin, Guglielmo Di Molfetta, Nicholas J. Ashton, Kaj Blennow, Henrik Zetterberg, Agneta K Nordberg

**Affiliations:** ^1^ Department of Neurobiology, Care Sciences and Society, Center for Alzheimer Research, Division of Clinical Geriatrics, Karolinska Institutet, Stockholm Sweden; ^2^ Theme Inflammation and Aging, Karolinska University Hospital, Stockholm Sweden; ^3^ Turku PET Centre, Turku University Hospital, University of Turku and Åbo Akademi University, Turku Finland; ^4^ Medical Radiation Physics and Nuclear Medicine, Section for Nuclear Medicine, Karolinska University Hospital, Stockholm Sweden; ^5^ Department of Neurology, Karolinska University Hospital, Stockholm Sweden; ^6^ ALZpath. Inc, Carlsbad, CA USA; ^7^ Department of Psychiatry and Neurochemistry, Institute of Neuroscience and Physiology, The Sahlgrenska Academy, University of Gothenburg, Mölndal Sweden; ^8^ Department of Psychiatry and Neurochemistry, Institute of Neuroscience and Physiology, The Sahlgrenska Academy, University of Gothenburg, Mölndal, Gothenburg Sweden; ^9^ Clinical Neurochemistry Laboratory, Sahlgrenska University Hospital, Mölndal Sweden; ^10^ Hong Kong Center for Neurodegenerative Diseases, Hong Kong China; ^11^ Wisconsin Alzheimer's Disease Research Center, University of Wisconsin School of Medicine and Public Health, Madison, WI USA; ^12^ UK Dementia Research Institute, University College London, London UK

## Abstract

**Background:**

Studies on plasma biomarkers have shown promising results for Alzheimer’s disease(AD) neuropathology in a research setting, but their associations to neuropsychological(NP) tests have not been extensively studied in clinical cohorts. The main aim was to investigate associations between NP, plasma biomarkers and amyloid accumulation in the brain(Aβ‐PET).

**Method:**

We quantified plasma biomarkers (tau phosphorylated at 217, 181 and 231, glial fibrillary acidic protein(GFAP) and neurofilament‐light(NFL)) by single molecular array in 126 patients (age=65±8) who were admitted to the Clinic for Cognitive Disorders, Karolinska University Hospital. Patients underwent an extensive initial clinical assessment encompassing MRI/CT, CSF sampling, NP evaluation and, within a year, a [18F]flutemetamol‐PET(Aβ‐PET) scan. NP assessment consisted of the following tests: RAVL(learning&retention), Rey‐Osterrieth(copy&retention), Trail making test(TMT) A&B and Digit Symbol. Test results were transformed in Z‐scores. At this point in the data collection, 47 patients were initially diagnosed as mild cognitive impaired(MCI) and had available >=1 of the NP tests. To refine the diagnosis, Aβ‐PET images were acquired and visually rated for positivity/negativity(VR) and quantified in Centiloid(Aβ‐PET‐CL) and after reassessment of the cohort, 28 subjects with available NP scores became MCI‐Aβ+(n=12) or Aβ‐(n=16). Significant(p<0.05) associations with Spearman’s coefficients(rho) are reported. Z‐scores were used to predict Aβ‐PET+ with receiver‐operating‐characteristic curves and area‐under‐curves(AUC).

**Result:**

Cognitive performance(Z‐scores) of the MCI cohort(after PET) was on average 1‐2 standard deviations below the reference database and Rey‐Osterrieth‐copy correlated with Rey‐Osterrieth‐retention(rho=0.44) and Digit Symbol(rho=0.43) tests. MCI‐Aβ+ and Aβ‐ groups did not statistically differ in cognitive performance. TMT‐A only presented an association with Aβ‐PET‐CL(rho=0.64, n=11)(Figure 1) and RAVL retention correlated negatively with plasma NFL(rho=0.46, n=26)(Figure 2); these correlations were significant in the Aβ+ group but not in the Aβ‐ group. In the MCI cohort before Aβ‐PET, TMT‐A correlated with plasma pTau231(rho=0.59, n=17)(Figure 3). From preliminary analyses, Z‐Scores seem to poorly predict VR(AUC<=0.65).

**Conclusion:**

The most promising results from TMT‐A need to be confirmed when all the data are available, TMT‐A was associated with plasma NFL and pTau231 and Aβ‐PET, but not with pTau217 and GFAP, previously found to be associated to Aβ‐PET. Poor performance of NP tests alone in predicting amyloid accumulation in the brain might require integration with other biomarkers.